# Hyponatremia—A New Diagnostic Marker for Complicated Acute Appendicitis in Children: A Systematic Review and Meta-Analysis

**DOI:** 10.3390/children9071070

**Published:** 2022-07-18

**Authors:** Sachit Anand, Nellai Krishnan, Jana Ròs Birley, Goran Tintor, Minu Bajpai, Zenon Pogorelić

**Affiliations:** 1 Department of Pediatric Surgery, All India Institute of Medical Sciences, New Delhi 110029, India; kanusachit@gmail.com (S.A.); nellai93@gmail.com (N.K.); bajpai2b@gmail.com (M.B.); 2 Department of Surgery, School of Medicine, University of Split, 21000 Split, Croatia; janabirley@gmail.com; 3 Department of Surgery, University Hospital of Split, 21000 Split, Croatia; gogitintor@gmail.com; 4 Department of Pediatric Surgery, University Hospital of Split, 21000 Split, Croatia

**Keywords:** hyponatremia, biomarkers, acute appendicitis, complicated appendicitis, children

## Abstract

Background: Acute appendicitis in the pediatric population remains a diagnostic challenge for clinicians. Despite many biochemical markers, imaging modalities and scoring systems, initial misdiagnosis and complication rates are high in children. This suggests the need for investigations directed towards new diagnostic tools to aid in the diagnosis. Recent studies have shown a correlation between serum sodium levels and complicated appendicitis. Although the exact reasons for hyponatremia in patients with complicated appendicitis are not known, there is persuasive data to support the role of pro-inflammatory cytokines such as IL-6 in the non-osmotic release of antidiuretic hormone. This meta-analysis aims to investigate all available data on hyponatremia as a diagnostic marker of complicated appendicitis in the pediatric population. Methods: The literature search was conducted by two independent investigators according to the Preferred Reporting Items for Systematic Reviews and Meta-Analyses (PRISMA) guidelines. The scientific databases (PubMed, EMBASE, Web of Science, and Scopus) were systematically searched for relevant studies using the keywords (hyponatremia) AND (appendicitis) AND (children). The methodological quality was assessed using a validated scale, and RevMan 5.4 software was utilized for pooled analysis. Results: Seven studies were included in the final meta-analysis, five of which were retrospective. A total of 1615 and 2808 cases were distributed into two groups: group A with complicated appendicitis and group B with uncomplicated acute appendicitis, respectively. The studies compared serum sodium levels of patients among the groups. Pooling the data demonstrated significantly lower serum sodium levels in children with complicated appendicitis vs. the non-complicated appendicitis (WMD: −3.29, 95% CI = −4.52 to −2.07, *p* < 0.00001). The estimated heterogeneity among the included studies was substantial and statistically significant (I^2^ = 98%, *p* < 0.00001). Conclusion: The results of the present meta-analysis indicate that hyponatremia has potential to be utilized as a biochemical marker in the diagnosis of complicated appendicitis in the pediatric population. However, well designed prospective diagnostic efficiency studies are essential to consolidate the association between hyponatremia and complicated acute appendicitis.

## 1. Introduction

Acute appendicitis is the most common acute abdominal condition in the pediatric population [1]. This patient group remains a diagnostic challenge to clinicians due to atypical symptoms and difficulty in history taking, especially in children <5 years of age [2]. Children tend to have a longer duration of symptoms and significantly higher rates of perforations, with perforation rates between 31.8% and 45.8% and even higher rates in pre-school children [3,4].

The evaluation of suspected appendicitis is guided by several diagnostic tools, such as clinical examination, scoring systems, biochemical testing and imaging modalities. The Alvarado score and the pediatric appendicitis score (PAS) are widely used today when appendicitis is suspected, but have higher accuracy in older children [5]. Recently, the appendicitis inflammatory response (AIR) score has been created to overcome shortcomings of the Alvarado score and the PAS. Recent studies showed higher sensitivity and specificity in comparison with the Alvarado score and PAS [6]. Certain biomarkers are routinely assessed, and elevated levels of CRP > 8 mg/L and WBC > 12 × 10^9^ have been linked to perforation of the appendix [3]. Although clinical examination, different scoring systems and laboratory markers are used to help establish the diagnosis of acute appendicitis, initial misdiagnosis rates are estimated to be 28–57% in 2- to 12-year-old children [2,7]. This contributes to diagnostic delays and mandates new objective biomarkers to aid accurate and prompt diagnosis.

Appendectomy has long been considered the standard of care for acute appendicitis, but recent studies have demonstrated the effectiveness of conservative management of uncomplicated appendicitis [8]. Management of complicated appendix requires urgent intervention and intravenous broad-spectrum antibiotics, and complications include abscess formation and diffuse peritonitis. Non-operative management of non-complicated acute appendicitis was more frequently applied in children during the COVID-19 pandemic [9].

Measuring serum sodium level is a low-cost test routinely performed in patients. Recent studies have investigated the potential role of hyponatremia as a diagnostic marker of complicated appendicitis, and found a significant difference in sodium levels in patients with complicated appendicitis vs. uncomplicated cases [10,11]. These studies conclude that sodium levels < 135 mmol/L have a role in diagnosing complicated appendicitis, as a supplement to the preoperative risk assessment [12,13]. The pathogenesis behind the association of complicated appendicitis and hyponatremia remains uncertain, but new evidence suggests IL-6 has a role in osmoregulation in intra-abdominal inflammation, leading to release of vasopressin [13].

This study was designed to investigate the role of hyponatremia as a diagnostic marker of complicated appendicitis in children.

## 2. Materials and Methods

### 2.1. Search Strategy

The literature search was conducted as per the Preferred Reporting Items for Systematic Reviews and Meta-Analyses (PRISMA) guidelines [14]. Two investigators (N.K. and S.A.) independently conducted the systematic search in the PubMed, EMBASE, Web of Science, and Scopus databases on 5 May 2022. The search keywords used were (hyponatremia) AND (appendicitis) AND (children). The total search records were then analyzed and the duplications were removed. Subsequently, the eligibility criteria were applied to screen the relevant studies.

### 2.2. Eligibility Criteria

The inclusion criteria were: All comparative studies depicting serum sodium levels in children aged less than 18 years with acute complicated appendicitis. Complicated appendicitis was defined as the presence of perforation, or gangrenous appendix, or an intra-abdominal abscess, or fecal peritonitis. The non-complicated appendicitis group consisted of patients with acute non-complicated appendicitis. The studies where data reporting was incomplete or where the outcomes of interest were not reported were excluded. Case reports, literature reviews, commentaries, editorials, conference abstracts, and opinion articles were also excluded (Appendix A, Table A1).

### 2.3. Data Extraction

After obtaining the search results, data synthesis was performed by two independent investigators (N.K. and S.A.) using Microsoft Excel spreadsheets. The extracted data from the included studies consisted of the first author’s name, publication year, the study design, sample size, the average age of the cohort, and the average ± standard deviation (SD) serum sodium levels in children of both the groups. Disagreements, if any, were settled by discussions and consensus with the senior author (Z.P.). During data extraction, children with complicated and non-complicated appendicitis were assigned to groups A and B, respectively.

### 2.4. Quality Assessment

An independent assessment of the methodological quality was performed by two investigators (NK and ZP) using the Downs and Black scale [15]. This validated 27-point scale has four domains of assessment with minimum and maximum scores of 0 and 32, respectively. On the basis of these scores, the risk of bias was graded as high (0–15), moderate (16–23) or low (>23). Kappa statistics were used to identify the level of inter-rater agreement regarding the risk of bias [16]. The degree of agreement could be graded as slight (0.00–0.20), fair (0.21–0.40), moderate (0.41–0.60), substantial (0.61–0.80), and almost perfect (0.81–1.00).

### 2.5. Statistical Analysis

All numerical data were depicted as mean ± SD. As the outcome of concern was continuous, mean differences (MD) were calculated for each included study. Subsequently, the weighted mean difference (WMD) was calculated by the inverse variance (IV) method. I^2^ statistics were applied for the analysis of heterogeneity among the included studies. A substantial heterogeneity was interpreted when I^2^ was >50%. In cases of substantial heterogeneity, the random-effects model was used. A value of *p* < 0.05 was considered statistically significant. During this systematic review, the quantitative analysis was performed using the RevMan 5.4 (Cochrane Collaboration, London, UK) software.

## 3. Results

### 3.1. Characteristics of the Included Studies

**Figure 1:**
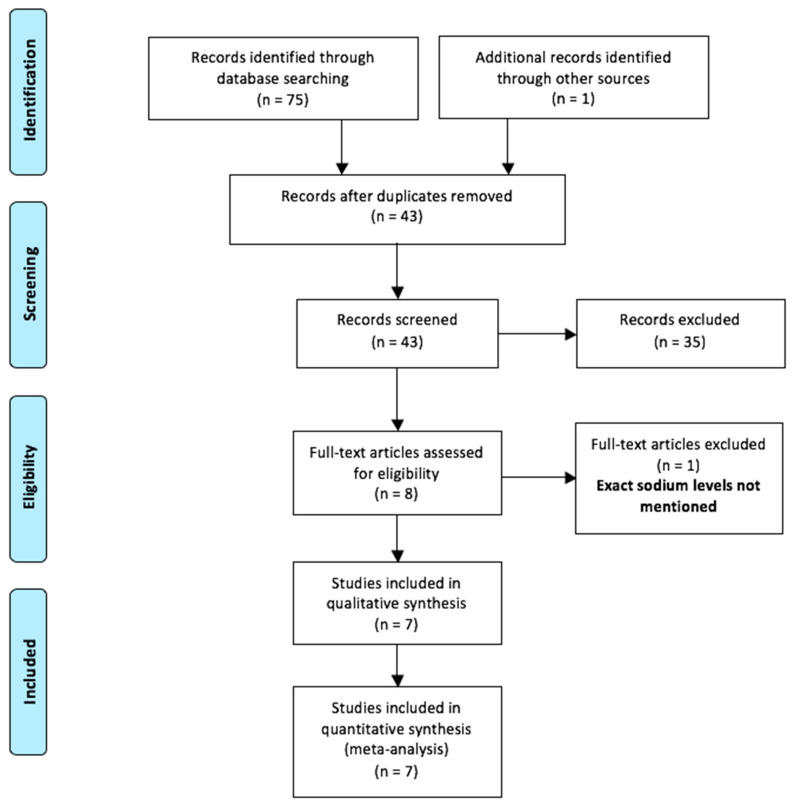
Selection of the relevant studies using the Preferred Reporting Items for Systematic Review and Meta-Analysis (PRISMA) flow diagram.

A total of seventy-six articles were identified with our search strategy (Annexure A). Of these, thirty-three duplicate records were eliminated (Figure 1). Out of forty-three remaining abstracts, thirty-five were excluded. Only eight full-texts were assessed for eligibility [10,11,17,18,19,20,21,22]. One of them was further excluded as it had not reported the exact sodium levels [22]. Therefore, only seven studies were included in the final meta-analysis [10,11,17,18,19,20,21]. Five of these seven studies had a retrospective study design [17,18,19,20,21]. The baseline characteristics of the included studies are depicted in Table 1. A male preponderance was observed in all studies. Various other biomarkers were explored in these studies, and are presented in Table 1.

Abbreviations: Retro—retrospective study; Pro—prospective study; Gp A—(group A), acute complicated appendicitis; Gp B—(group B), acute non-complicated appendicitis; CRP—C-reactive protein; WBC—white blood cell count; BE—Base excess; NP—neutrophil percentage; MLR—monocyte-to-lymphocyte ratio; NLR—neutrophil-to-lymphocyte ratio; PLR—platelet-to-lymphocyte ratio; MPV—mean platelet volume; AVP—arginine vasopressin; PC—platelet count; PCT—procalcitonin; DLAC—D-lactate; AST—aspartate aminotransferase; ALT—alanine aminotransferase.

### 3.2. Summary of the Included Studies

Pham et al., 2016. This retrospective study was conducted in the United States. A total of 392 patients aged <12 years with appendicitis were analyzed, of whom 179 (46%) had complicated appendicitis. The study showed that hyponatremia (OR = 3.1, 95%CI = 2.0–4.9, *p* < 0.01) was an independent diagnostic marker of complicated appendicitis. The median age in the group of patients with complicated appendicitis was 8 years, and in the non-complicated group, the median age was 9 years. Patients with complicated appendicitis had a significantly lower serum sodium level compared with non-complicated appendicitis (134 mEq/L vs. 137 mEq/L, *p* < 0.01). The study also showed that symptom duration > 24 h and leukocytosis were predictors of complicated appendicitis 17.

Besli et al., 2019. This retrospective study conducted in Turkey evaluated a total of 403 patients with acute appendicitis. The mean age in a group of patients with complicated appendicitis was 11.4 years, and in the non-complicated group, the median age was 11.3 years. Of these, 158 (39.2%) had non-complicated and 245 (60.8%) had complicated appendicitis. No difference was found between the two groups with regard to hyponatremia, leukocytosis, and neutrophilia (*p* > 0.05). However, patients with complicated appendicitis had lower baseline serum Na levels (*p* = 0.004; *p* < 0.05). For the diagnosis of complicated appendicitis, the cut-off value for Na was ≤ 138 mEq/L (sensitivity 82.5% and specificity 31.1%). 18.

Lindestam et al., 2019. This prospective study was conducted in Sweden. Eighty children with acute appendicitis (AA) confirmed on histopathology were included. The median age in the group of patients with complicated appendicitis was 7.5 years, and in the non-complicated group, the median age was 9.2 years. The median plasma sodium concentration on admission in patients with complicated AA (134 mmol/L, IQR 132–136) was significantly lower than in children with non-complicated AA (139 mmol/L, IQR 137–140). The receiver operating characteristic curve of plasma sodium concentration identifying patients with complicated AA showed an area under the curve of 0.93 (95% CI = 0.87–0.99), with a sensitivity and specificity of 0.82 (0.70–0.90) and 0.87 (0.60–0.98), respectively 11.

Yang et al., 2019. This retrospective study from China on 1892 children aged between 3 to 18 years with confirmed acute appendicitis showed significantly lower mean serum sodium levels in complicated appendicitis compared with non-complicated appendicitis (133 mEq/L vs. 137 mEq/L, *p* = 0.001). The median age in the group of patients with complicated appendicitis was 5 years, and the in non-complicated group, the median age was 9 years. The study also highlighted white blood cell count, C-reactive protein and neutrophils percentage as important markers in distinguishing complicated appendicitis from non-complicated appendicitis 19.

Pogorelić et al., 2021. This prospective study conducted in Croatia aimed to investigate hyponatremia as a new biochemical marker associated with complicated appendicitis. A total of 184 children with histopathologically confirmed acute appendicitis were enrolled, of whom 146 patients and 38 patients had non-complicated and complicated appendicitis, respectively. The median age in the group of patients with complicated appendicitis was 10.4 years, and in the non-complicated group, the median age was 11.6 years. The study found that the mean serum sodium level in patients with complicated appendicitis was significantly lower compared with patients with non-complicated appendicitis (132.2 mmol/L vs. 139.2 mmol/L, *p* < 0.001). The study also found that a cut-off-value of plasma sodium concentration of ≤135 mmol/L was shown to give the best possible sensitivity and specificity, 94.7% (95% CI: 82.2–99.3) and 88.5% (95% CI: 88.2–93.2), respectively, (*p* < 0.001) 10.

Duman et al., 2022. In this retrospective study from Turkey, a total of 683 children were included. The mean age in the group of patients with complicated appendicitis was 9.4 years, and in the non-complicated group, the median age was 10.2 years. The cohort included children with acute appendicitis (AA, *n* = 254), complicated appendicitis (PA, *n* = 82), nonspecific abdominal pain (NAP, *n* = 197), and controls (*n* = 150). This study showed that serum sodium levels were significantly decreased in patients with AA (*p* < 0.05). A cut-off serum sodium of < 137 mmol/L could identify appendicitis with sensitivity of 72% and specificity of 42%. However, there was no significant difference between the AA and PA groups in terms of serum sodium levels 20.

Walsh et al., 2022. This retrospective study was conducted in New Zealand. A total of 1283 pediatric patients (≤15 years) underwent appendectomy, of whom 443 (35%) had complicated appendicitis, 690 (54%) had non-complicated appendicitis, and 26 (3.8%) had no appendicitis. The median age in a group of patients with complicated appendicitis was 10 years, and in the non-complicated group, the median age was 11.5 years. A significant difference was observed among the three patient groups in terms of the serum sodium levels. Hyponatremia was seen in 31.4% of the complicated group, 3.8% of the non-complicated group, and 10.7% of the no appendicitis group. A cut-off serum sodium of <135 mmol/L could identify complicated appendicitis with a sensitivity of 31.4% and a specificity of 95.7% 21.

### 3.3. Methodological Quality Assessment

The detailed quality assessment by two independent authors is depicted in Table 2. As per the Downs and Black scale, the average scores assigned to the included studies ranged from 24 to 27.5. All the studies had a low risk of bias. While the study by Pogorelić et al. [10] had the minimum risk of bias (score = 27.5), the retrospective study by Pham et al. [17] had a score of 24, the minimum out of all studies. The inter-observer agreement was almost perfect (Kappa = 0.91, *p* < 0.0001).

### 3.4. Outcome Analysis

**Figure 2:**
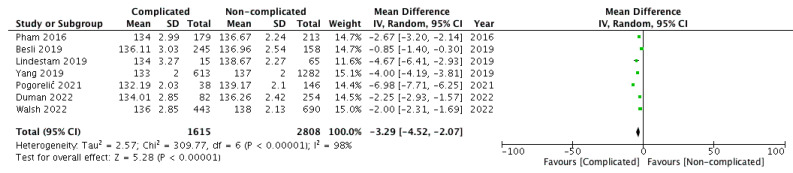
Forest plot comparison of serum sodium levels between the two patient groups, i.e., children with complicated (group A) versus non-complicated acute appendicitis (group B). Abbreviations: SD—standard deviation; IV—inverse variance; CI—H interval.

The outcome of interest was reported by all seven included studies [4,5,6,7,8,9,10]. The serum sodium levels were compared between 1615 and 2808 children belonging to groups A and B, respectively. Pooling the data (Figure 2) demonstrated significantly lower serum sodium levels in children within group A versus group B (WMD: −3.29, 95% CI = −4.52 to −2.07, *p* < 0.00001). For this outcome, the estimated heterogeneity among the included studies was substantial and statistically significant (I^2^ = 98%, *p* < 0.00001).

## 4. Discussion

Acute appendicitis is the most common abdominal surgical emergency in the pediatric population [23,24]. Approximately 20–30% of children with acute abdominal pain admitted to pediatric surgical departments are diagnosed with acute appendicitis [25]. Despite being a relatively common condition, acute appendicitis is still a cause of diagnostic quandary for clinicians and frequently presents with atypical symptoms [6]. Children tend to have significantly higher rates of perforation compared with adults, especially those under 5 years of age [3,26,27,28,29]. The standard of treatment in the majority of the centers worldwide is still laparoscopic appendectomy, although recently non-complicated forms of acute appendicitis are more frequently managed with a non-operative approach [8,9,27,30,31,32]. Postponed diagnosis and treatment often increase the risk of other complications, such as abscess formation, peritonitis and partial bowel obstruction [27,32].

Clinical and laboratory data have been combined in the form of different clinical scoring systems which contribute satisfactory general sensitivity, but specificity and consequently the ability to precisely diagnose acute appendicitis is below expectations [5,6,33,34,35]. A large assortment of diagnostic procedures, for example CT scans and ultrasonography, are currently available and exhibit high sensitivity and specificity rates even though their utilization is significantly limited by multifarious factors, such as immediate availability, expenses, ionizing radiation with associated risk of developing cancer, especially for pediatric patients, and the national guidelines [35,36]. Recent studies have attempted to identify available, easily detected and relatively inexpensive biomarkers not only to confirm the presence of acute appendicitis but also to predict complicated acute appendicitis.

Hyponatremia at hospital admission has been established as a diagnostic marker of gangrenous cholecystitis, ischemic bowel in patients presenting with a mechanical small bowel obstruction and perforation of the large bowel in elderly patients who have undergone emergency general surgery [37,38,39]. Hyponatremia has also been associated with increased mortality in patients with necrotizing soft-tissue infections and has been recognized as an instrument to differentiate necrotizing soft-tissue infections from a variety of other infections [40,41]. Furthermore, preoperative hyponatremia can be used independently from standard risk factors to distinguish high risk patients for cardiac surgery [42,43,44]. Zhang et al. in their study suggested hyponatremia as a notable and potentially clinically applicable point of reference regarding intra-abdominal sepsis and anastomotic leakage in patients following colorectal surgery [45]. In addition, among hospitalized patients, the presence of hyponatremia directly corresponds to increased morbidity, extended lengths of stay and greater utilization costs [46,47,48,49,50,51]. Although the accurate etiology for hyponatremia in patients with complicated appendicitis is still not identified, there is persuasive data to support a role for pro-inflammatory cytokines, such as IL-6, IL-1β, etc., in the non-osmotic release of antidiuretic hormone (ADH). The circulating cytokines cross the blood-brain barrier and act on the neurons of the supraoptic and the paraventricular nucleus. Subsequently, there is activation of the Janus tyrosine kinases-signal transducer and activator of transcription (JAK-STAT) pathway, thus leading to the release of ADH [52,53,54,55,56]. This non-osmotic release of ADH leads to excess free water reabsorption in the kidneys and causes dilutional hyponatremia. Some recently published studies investigated hyponatremia as a novel diagnostic marker of complicated appendicitis [10,11,17,18,19,20,21].

Seven studies associating hyponatremia and complicated appendicitis were included in this meta-analysis, and statistically significant results were found in five of them. Furthermore, five of these seven studies were designed as retrospective studies and two of them were prospective. Regardless of being retrospective, the study by Yang at al. had the largest sample size (1895 children) and demonstrated that elevated levels of neutrophil percentage (>74%) and CRP (>8 mg/dL) combined, increased the risk of complicated appendicitis more than five times [19]. It has been suggested by additional studies that in order to strengthen their sensitivity and specificity, hyponatremia should be considered concurrently with WBC and CRP to achieve the most accurate outcome in terms of differentiating complicated appendicitis from non-complicated appendicitis [3]. On the other hand, Pham et al. provided substantial evidence in numerous logistic regression analyses (OR = 3.1, 95% CI = 2.0–4.9, *p* < 0.01) indicating that hyponatremia was an independent prognosticator of complicated appendicitis [17]. Moreover, Walsh et al. firmly corroborated this in their large-scale retrospective review [21].

The literature is nevertheless inconsistent with Duman et al. and Besli et al. presenting no correlation between the hyponatremia and complicated appendicitis in the pediatric population. For the diagnosis of complicated appendicitis, Duman et al. and Besli et al. designated a cut-off value for serum sodium of <137 mmol/L and ≤138 mmol/L, respectively [18,20]. Several other laboratory markers have recently been investigated, such as hyperbilirubinemia, MPV, RDW, and interleukins, but none of these have the same levels of sensitivity and specificity as hyponatremia in the detection of complicated appendicitis [57,58,59,60].

The prospective studies were found to make a larger contribution by consolidating previously determined reports of statistically significantly increased values of hyponatremia in children with complicated appendicitis in contrast to non-complicated appendicitis. Both Pogorelic et al. and Lindestam et al. reported that patients, who were sampled at the pediatric emergency department but later excluded from their final analyses as they did not have appendicitis verified by histopathology, had similar median sodium concentrations to those with non-complicated appendicitis [10,11].

The results of this meta-analysis should be considered carefully within the context of several limitations. First, there are several areas of concern within the current literature. The study by Walsh et al. [21] had missing data and also included only patients who underwent surgery for suspected appendicitis, rather than all patients who were admitted to the pediatric emergency department with clinical suspicion of appendicitis. This patient selection process should be considered as a factor impacting the strength of the study. Additionally, a non-uniformity in the selection of cut-off values of hyponatremia was observed among the included studies which may influence the generalizability of the findings. Hyponatremia was defined as plasma sodium concentration ≤135 mmol/L in three studies, whereas the remaining four studies defined hyponatremia at a level of <135 mmol/L, ≤136 mmol/L, <137 mmol/L or ≤138 mmol/L, respectively. It is suggested that in further research, the cut-off value of ≤135 mmol/L should be used considering the normal required minimum serum sodium level. Second, these studies only considered the association of hyponatremia with macroscopic pathological change without investigating the association between the degree of hyponatremia severity and histopathological changes. Third, five out of the seven studies had a retrospective study design. Finally, it is imperative to explore the accuracy of a panel of biomarkers rather than focusing on only one biomarker. It will be interesting to study the combined sensitivity and specificity of a panel of biomarkers including serum sodium, serum fibrinogen, and serum bilirubin in well-designed prospective studies as all of these have shown an association with complicated appendicitis.

## 5. Conclusions

To conclude, the results of this meta-analysis favor the identification of serum sodium level as an easily conducted, low-cost laboratory test, which should be taken into consideration in children with a suspicion of acute appendicitis and underlying complications. However, well-designed prospective diagnostic efficiency studies are essential to consolidate the association between hyponatremia and complicated acute appendicitis.

## Figures and Tables

**Table 1:** Baseline characteristics of the included studies.

Author	Study Design	Sample Size		Gender (% Males)		Other Biomarkers Investigated
		Gp A	Gp B	Gp A	Gp B	
Pham et al., 2016 [17]	Retro	179	213	63%	69%	WBC
Besli et al., 2019 [18]	Retro	245	158	70%	64%	WBC, NP, CRP
Lindestam et al., 2019 [11]	Pro	15	65	80%	63%	CRP, WBC, plasma glucose, BE, plasma AVP
Yang et al., 2019 [19]	Retro	613	1282	54%	52%	CRP, WBC, NP, PC, PCT, DLAC, Bilirubin, AST, ALT
Pogorelić et al., 2021 [10]	Pro	38	146	71%	63%	WBC, CRP, NP, potassium, chloride, glucose
Duman et al., 2022 [20]	Retro	82	254	2:1 *		CRP, WBC, NP, MLR, NLR, PLR, MPV
Walsh et al., 2022 [21]	Retro	443	690	60%	61.4%	-

* Group-wise gender distribution not mentioned. In this study, M:F ratio among the appendicitis group was 2:1.

**Table 2:** Independent methodological quality assessment by two observers utilizing the Downs and Black scale.

Study	Reporting	External Validity	Internal Validity-Bias	Internal Validity- Confounding	Power	Total Scores
Quality assessment by observer 1						
Pham et al., 2016 [17]	7	3	5	3	5	23
Besli et al., 2019 [18]	9	3	4	3	5	24
Lindestam et al., 2019 [11]	10	3	5	3	5	26
Yang et al., 2019 [19]	10	3	4	3	5	25
Pogorelić et al., 2021 [10]	11	3	5	4	5	28
Duman et al., 2022 [20]	9	3	5	3	5	25
Walsh et al., 2022 [21]	9	3	5	3	5	25
Quality assessment by observer 2						
Pham et al., 2016 [17]	9	3	5	3	5	25
Besli et al., 2019 [18]	9	3	5	3	5	25
Lindestam et al., 2019 [11]	11	3	4	4	5	27
Yang et al., 2019 [19]	11	3	4	4	5	27
Pogorelić et al., 2021 [10]	11	3	4	4	5	27
Duman et al., 2022 [20]	9	3	5	3	5	25
Walsh et al., 2022 [21]	9	3	4	3	5	24
Total scores and inter-observer agreement						
Study	Rater 1	Rater 2	Mean	Kappa value	*p*	
Pham et al., 2016 [17]	23	25	24	0.91	<0.0001	
Besli et al., 2019 [18]	24	25	24.5			
Lindestam et al., 2019 [11]	26	27	26.5			
Yang et al., 2019 [19]	25	27	26			
Pogorelić et al., 2021 [10]	28	27	27.5			
Duman et al., 2022 [20]	25	25	25			
Walsh et al., 2022 [21]	25	24	24.5			

## Data Availability

The data presented in this study is available upon request to the authors.

## Appendix A

PubMed: (hyponatremia) AND (appendicitis) AND (children)
Embase: (‘hyponatremia’/exp OR hyponatremia) AND (‘appendicitis’/exp OR appendicitis) AND (‘child’/exp OR child)
Scopus: (TITLE-ABS-KEY (hyponatremia) AND TITLE-ABS-KEY (appendicitis) AND TITLE-ABS-KEY (children))
Web of Science: Query 1: ALL = (hyponatremia) AND Query 2: ALL = (appendicitis) AND Query 3: (children)

**Table A1:** Results of the search strategy.

Database	Studies
PubMed	11
Embase	25
Scopus	26
Web of Science	13
Additional records from other sources	01
Total	76
Duplications	33
After duplications removal	43
